# Intracellular phase for an extracellular bacterial pathogen: MgtC shows the way

**DOI:** 10.15698/mic2015.09.227

**Published:** 2015-08-13

**Authors:** Audrey Bernut, Claudine Belon, Chantal Soscia, Sophie Bleves, Anne-Béatrice Blanc-Potard

**Affiliations:** 1 Laboratoire de Dynamique des Interactions Membranaires Normales et Pathologiques, Université de Montpellier (DIMNP CNRS-UMR5235), Place Eugène Bataillon, 34095 Montpellier Cedex 05, France.; 2 CNRS & Aix-Marseille University, Laboratoire d’Ingénierie des Systèmes Macromoléculaires (UMR7255), IMM, 31 Chemin Joseph Aiguier, 13402 Marseille cedex 20, France.

**Keywords:** Pseudomonas aeruginosa, MgtC, macrophage, magnesium, biofilm, zebrafish

## Abstract

* Pseudomonas aeruginosa* is an extracellular pathogen known to impair host phagocytic functions. However, our recent results identify MgtC as a novel actor in* P. aeruginosa* virulence, which plays a role in an intramacrophage phase of this pathogen. In agreement with its intracellular function, *P. aeruginosa* *mgtC* gene expression is strongly induced when the bacteria reside within macrophages. MgtC was previously known as a horizontally-acquired virulence factor important for multiplication inside macrophages in several intracellular bacterial pathogens. MgtC thus provides a singular example of a virulence determinant that subverts macrophages both in intracellular and extracellular pathogens. Moreover, we demonstrate that *P. aeruginosa* MgtC is required for optimal growth in Mg^2+ ^deprived medium, a property shared by MgtC factors from intracellular pathogens and, under Mg^2+ ^limitation,* P. aeruginosa* MgtC prevents biofilm formation. We propose that MgtC has a similar function in intracellular and extracellular pathogens, which contributes to macrophage resistance and fine-tune adaptation to the host in relation to the different bacterial lifestyles. MgtC thus appears as an attractive target for antivirulence strategies and our work provides a natural peptide as MgtC antagonist, which paves the way for the development of MgtC inhibitors.

There is growing evidence that pathogens recognized as extracellular can actually survive inside macrophages after phagocytosis to hide from the immune system and spread, but little is known about the intracellular behavior of these pathogens and about the bacterial factors involved. For example, *Staphylococcus aureus* has long been considered as an extracellular pathogen, but infections in the zebrafish embryo model have contributed to reveal an intracellular phase in phagocytic cells in *S.* *aureus* life cycle. *P. aeruginosa* is known to impair host phagocytic functions, mainly by using cytotoxic and anti-phagocytic determinants. Unexpectedly, our recent work revealed a novel bacterial factor implicated in a macrophage intracellular stage during *P. aeruginosa* acute infection.

Common strategies can be used by bacterial pathogens that share similar lifestyle or similar environmental niches. MgtC is a virulence factor common to several intracellular pathogens including *Salmonella enterica *serovar Typhimurium (*S. *Typhimurium) and *Mycobacterium tuberculosis*. Importantly, MgtC has been shown to promote intramacrophage survival of *Yersinia pestis*, which is a facultative intracellular pathogen that replicates mainly extracellularly and produces anti-phagocytic factors. In *Salmonella*, MgtC promotes pathogenicity by inhibiting the bacterial F_1_F_o_ ATP synthase. *Salmonella* *mgtC* is regulated both at the transcriptional and post-transcriptional levels, with a positive regulation by Mg^2+^ deprivation and increase in cytosolic ATP, and a negative regulation by the MgtR peptide. In agreement with the Mg^2+^ regulation, MgtC has been involved in adaptation to low Mg^2+^ environments.

The environmental bacterium and opportunistic human pathogen *P. aeruginosa* is a major cause of mortality in cystic fibrosis (CF) patients. Interestingly, *mgtC* has been highlighted as a horizontally-acquired gene shared by several opportunistic bacteria infecting CF patients, including *P. aeruginosa*, *Burkholderia ceanocepacia* and *Mycobacterium abscessus*. MgtC exhibits a sporadic distribution in *Pseudomonas* species, being mostly associated with strains that are pathogenic for humans and insects. Our phylogenetic analysis indicated that the MgtC protein from the extracellular pathogen *P. aeruginosa* clustered in the same subgroup as MgtC proteins from intracellular pathogens. We investigated the role of *P. aeruginosa* MgtC by analyzing the infection phenotypes and behavior in Mg^2+^ deprived conditions of an *mgtC* mutant.

To evaluate the role of MgtC in *P. aeruginosa* virulence, we used the zebrafish (*Danio rerio) *embryo model, which is a model of choice to investigate the contribution of cells from the innate immune system during infection. This model possesses professional phagocytes that can engulf and kill *P. aeruginosa* upon a systemic infection. We showed that the *P. aeruginosa* *mgtC* mutant is attenuated for acute infection in zebrafish embryos. MgtC most likely acts by protecting *P. aeruginosa* against phagocytes since macrophage depletion (using *pu.1* morpholinos) suppressed the difference between *mgtC* mutant and wild-type PAO1 strain. This hypothesis was supported by *ex vivo* experiments since the *mgtC* mutant is more phagocytosed and more sensitive to bacterial killing than the wild-type strain in J774 macrophages. Hence, similarly to intracellular pathogens, the MgtC virulence determinant of *P. aeruginosa* plays a role towards macrophages. We propose that *P. aeruginosa* has acquired a macrophage subversion factor to resist killing in case of phagocytosis by macrophages and further studies will be required to explore the fate of intracellular bacteria.

In agreement with the intramacrophage role of *P. aeruginosa *MgtC, expression of the *mgtC* gene is highly induced when the bacteria reside inside macrophages. To our knowledge, this is the first report of a *P. aeruginosa* gene induced within phagocytic cells. We further demonstrated that phagosome acidification contributes to an optimal expression of *Pseudomonas* *mgtC*. A strong intramacrophage induction has also been reported for the *Salmonella* *mgtC* gene. The intracellular signal that induces *Salmonella mgtC* transcription is still a matter of debate, but one hypothesis is a combination of cationic peptides and acidic pH. An open task is to uncover the molecular basis for the conservation of *mgtC* regulation among different species.

We established that *mgtC* expression in *P. aeruginosa* is induced in conditions of Mg^2+^ starvation, which is also the case for *mgtC* genes from intracellular pathogens. Growth kinetic analyses showed that MgtC is required for optimal growth in Mg^2+^ deprived medium in *P. aeruginosa*, another common feature of intracellular pathogens. Our results thus indicate a mechanism of convergent evolution between pathogens in their ability to use and regulate MgtC to adapt to Mg^2+ ^limitation. We speculate that this feature does not account for the role of *P. aeruginosa* MgtC in macrophages because addition of extracellular Mg^2+ ^did not rescue the increased sensitivity of *mgtC* mutants to macrophages.

*P. aeruginosa* virulence and resistance to treatment is largely due to its ability to form biofilms, whose formation/stability can be affected by extracellular cations, including magnesium ions. Bacterial adherence to glass was visualized and quantified using crystal violet staining to infer the ability of strains to form biofilms. We observed that MgtC expression limits biofilm formation under Mg^2+^ limiting conditions. The biofilm phenotype of the *mgtC* mutant is associated with increased production of exopolysaccharides (EPS), which are essential biofilm matrix components. Interestingly, a recent report of Groisman’s laboratory provides an unsuspected link between MgtC, cellulose and intramacrophage survival in *Salmonella* since cellulose production, which is repressed by MgtC in low Mg^2+ ^medium, was shown to impede *Salmonella* replication inside macrophages. Cellulose is not produced by *P. aeruginosa* strains, but our finding of an increased EPS production by the *P. aeruginosa* *mgtC* mutant in low Mg^2+ ^medium, suggests a potential link between EPS production and the role of *P. aeruginosa* MgtC in macrophages. Further studies will be required to determine the nature of the EPS involved, to investigate their production intracellularly and the putative link to intramacrophage phenotypes.

**Figure 1:**
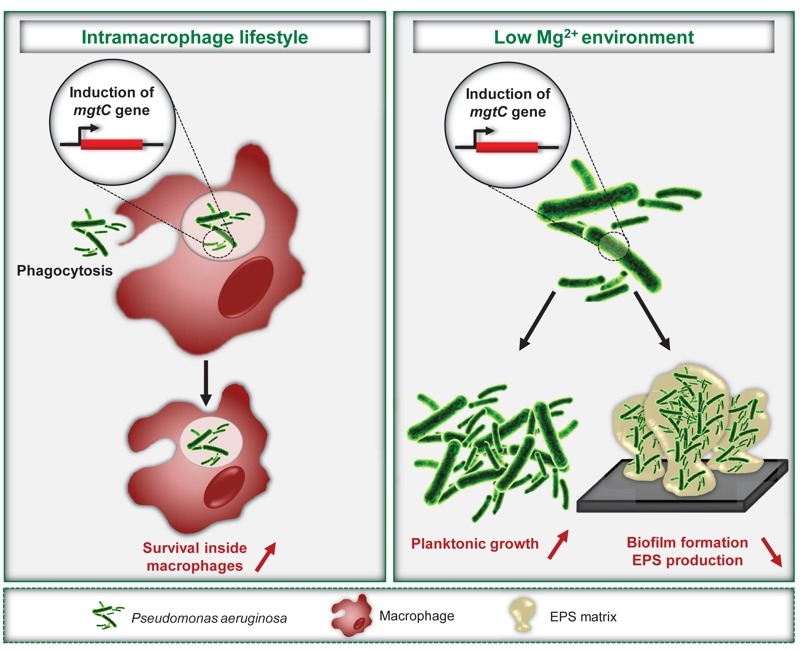
FIGURE 1: *P. aeruginosa* MgtC promotes an intramacrophage phase and limits biofilm formation. Expression of *P. aeruginosa* *mgtC* gene is induced inside macrophages and in magnesium-deprived medium. *P. aeruginosa* MgtC is involved in resistance to macrophage, a feature shared with MgtC proteins from intracellular pathogens as *S.* Typhimurium. Under magnesium deprivation, MgtC promotes planktonic growth but limits biofilm formation and EPS production.

Our findings highlight the importance of macrophage subversion by *P. aeruginosa* in a model of acute infection. Being a determinant that plays a role during infection both in intracellular and extracellular pathogens, MgtC appears as a promising new target for antivirulence strategies. We have proposed the MgtR membrane peptide (issued from *Salmonella*) as a natural MgtC antagonist because in *S. *Typhimurium, the intramacrophage replication of a wild-type strain can be reduced upon over-production of MgtR, which plays a negative regulatory role on MgtC expression. *P. aeruginosa* does not encode any MgtR homologue but in a bacterial two-hybrid system, MgtR interacts with both *S. *Typhimurium and *P. aeruginosa* MgtC. We have found that the phenotypes observed with *Pseudomonas* *mgtC* mutant in animal and cellular infection models can be mimicked upon heterologous production of the *Salmonella* MgtR peptide in a wild-type *P. aeruginosa* strain. Our results thus support an action of MgtR peptide as an antagonist of MgtC and paves the way for the development of antivirulence strategies based on MgtC inhibition.

